# BPDA - A Bayesian peptide detection algorithm for mass spectrometry

**DOI:** 10.1186/1471-2105-11-490

**Published:** 2010-09-29

**Authors:** Youting Sun, Jianqiu Zhang, Ulisses Braga-Neto, Edward R Dougherty

**Affiliations:** 1 Department of Electrical and Computer Engineering, Texas A&M University, College Station, TX 77843, USA; 2 Department of Electrical and Computer Engineering, University of Texas at San Antonio, San Antonio, TX 78249, USA; 3 Computational Biology Division, Translational Genomics Research Institution, Phoenix, AZ 85004, USA; 4 Department of Bioinformatics and Computational Biology, University of Texas M.D. Anderson Cancer Center, Houston, TX 77030, USA

## Abstract

**Background:**

Mass spectrometry (MS) is an essential analytical tool in proteomics. Many existing algorithms for peptide detection are based on isotope template matching and usually work at different charge states separately, making them ineffective to detect overlapping peptides and low abundance peptides.

**Results:**

We present BPDA, a Bayesian approach for peptide detection in data produced by MS instruments with high enough resolution to baseline-resolve isotopic peaks, such as MALDI-TOF and LC-MS. We model the spectra as a mixture of candidate peptide signals, and the model is parameterized by MS physical properties. BPDA is based on a rigorous statistical framework and avoids problems, such as voting and ad-hoc thresholding, generally encountered in algorithms based on template matching. It systematically evaluates all possible combinations of possible peptide candidates to interpret a given spectrum, and iteratively finds the best fitting peptide signal in order to minimize the mean squared error of the inferred spectrum to the observed spectrum. In contrast to previous detection methods, BPDA performs deisotoping and deconvolution of mass spectra simultaneously, which enables better identification of weak peptide signals and produces higher sensitivities and more robust results. Unlike template-matching algorithms, BPDA can handle complex data where features overlap. Our experimental results indicate that BPDA performs well on simulated data and real MS data sets, for various resolutions and signal to noise ratios, and compares very favorably with commonly used commercial and open-source software, such as flexAnalysis, OpenMS, and Decon2LS, according to sensitivity and detection accuracy.

**Conclusion:**

Unlike previous detection methods, which only employ isotopic distributions and work at each single charge state alone, BPDA takes into account the charge state distribution as well, thus lending information to better identify weak peptide signals and produce more robust results. The proposed approach is based on a rigorous statistical framework, which avoids problems generally encountered in algorithms based on template matching. Our experiments indicate that BPDA performs well on both simulated data and real data, and compares very favorably with commonly used commercial and open-source software. The BPDA software can be downloaded from http://gsp.tamu.edu/Publications/supplementary/sun10a/bpda.

## Background

Mass spectrometry (MS) is a key analytical tool in proteomics. A mass spectrometer measures the concentration of ionized molecules at a range of mass-to-charge ratios (*m/z*). MS instruments consist of three modules: an ionization source, a mass analyzer and a detector which captures the ions and measures the intensity of each ion species. Widely used ionization methods include electrospray ionization (ESI) [1] and matrix-assisted laser desorption/ionization (MALDI) [2,3]. Mass analyzers separate the ions according to their mass-to-charge ratios. There are several types of mass analyzers including the Orbitrap [4], Quadrupole [5], Time-of-Flight (TOF) [6,7], and fourier transform ion cyclotron resonance (FTICR) [8]. Liquid Chromatography (LC) is often coupled with MS to achieve additional separation of peptides and thus reduce the complexity of an individual mass spectrum. Before entering the mass spectrometer, peptide species pass through a LC column with different speeds depending on their physicochemical properties and interactions with the solvent [9]. A single LC-MS experiment usually produces hundreds to thousands of mass spectra sampled during the LC elution process.

Peptide detection, which converts raw spectra to a list of peptide masses, is usually the first step in protein MS data processing. It directly affects the accuracy of subsequent analyses such as protein identification and quantification, data alignment between multiple experiments, biomarker discovery and classification of different samples. One difficulty in peptide detection is that a peptide species may register several peaks in the spectra due to the following two points: First, a peptide species may take different numbers of charges during ionization, therefore its peaks can be observed at different charge states. Second, at a given charge state, several peaks can be observed due to heavy isotopes (e.g. ^13^*C*), which are commonly referred to as isotopic peaks or the isotope series. The isotopic distribution of a peptide can be determined by the elemental formula of the peptide and the natural abundance of heavy isotopes, and therefore known [10]. When mass spectra have low resolution in which isotopic peaks cannot be baseline resolved (i.e. the isotopic peaks convolve together to form isotope envelopes, and only one peak can be observed for one peptide at a given charge state), and when peptides are singly charged as commonly observed in MALDI, to report each detected peak as a peptide feature might be sufficient as in [11-14]. But for high resolution spectra, reporting each observed peak as a unique peptide species would give rise to too many false positives. Thus a variety of algorithms for deisotoping and charge states deconvolution have been proposed. Many of these algorithms such as PepList [15], msInspect [16], Noy's method [17], Decon2LS [18], and OpenMS [19] are based on template matching. Templates employed in the first four algorithms are based on 1 D theoretic isotope patterns predicted from peptide masses [20-22], while the last algorithm combines isotope patterns (along the *m/z *dimension) with elution peaks (along the elution time dimension) to form 2 D templates. If the observed signal (a cluster of peaks) matches the proposed template well -- the quality of the match is assessed by a fitting score -- it will be reported as a feature and then subtracted from the spectrum. The process iterates until no more matches can be found. The major problem of this matching and subtraction process is that it may be ineffective to detect overlapping peptides. In the case of overlapping (e.g. one doubly charged peptide can overlap with a singly charged peptide of half the mass), if the peak cluster of one peptide is incorrectly matched and subtracted, the rest of the peptides can not be detected correctly based on the remaining spectrum, which will cause error propagation. Besides, each predicted template is based on a single peptide and it can not match the observed overlapping peaks well, which renders a low quality match and reduces the sensitivities of these algorithms. In addition to these algorithms based on template matching, Du *et al*. developed an algorithm based on variable selection [10]. The key idea is to select the least number of candidate isotope series to explain the spectrum, and hence find the corresponding peptides. But the superimposed criterion "selecting the least number of candidates" is not justified. In fact, while this criterion may result in a desirable reduction of the false detection rate, the sensitivity may be reduced as well. Zhang *et. al*. [23] proposed a Bayesian approach for peptide ion peak detection. A model for the *m/z *interval of one dalton was developed, a Bayesian approach was applied to estimate the model parameters based on the observed spectrum, and the existence probability of a peptide ion peak at each charge state and isotope position was calculated. Note that this algorithm did not perform peptide level peak detection as what we are proposing in this paper. The authors reported that their method had better sensitivity results than the wavelet based algorithms when tested by simulated data and eight sets of real prOTOF MS data. In this paper, we propose a Bayesian Peptide Detection Algorithm (BPDA), which is basically an extension of Zhang's method. The difference being that a model for the whole spectrum is developed, and both isotope patterns and charge state distributions of peptides are considered in our method.

The proposed method, BPDA, can be applied to data generated by MS instruments with mass resolutions high enough to baseline-resolve isotopic peaks. BPDA evaluates all possible combinations of possible peptide candidates (originated from well-defined peaks of the raw spectrum -- see Methods section for more details) to interpret a given spectrum, and iteratively finds the best fitting peptide parameters (peptide peak heights, existence probabilities, etc.) in order to minimize the mean squared error of the inferred spectrum to the observed spectrum. BPDA offers the following four advantages: Firstly, since BPDA looks for the optimal among all possible interpretations of the MS spectra, the procedure is thus systematic. In contrast, the aforementioned template-matching methods are greedy. They cannot evaluate all potential interpretations of a given spectrum. Hence, they are neither systematic nor optimal. Besides, many methods do not include all possible peptide candidates, especially the ones with low abundance in the first place, and the sensitivity is reduced compared to BPDA. Secondly, BPDA considers all charge states and isotopic peaks of peptides for detection. It is noted that multiply charged peptides can register peaks at several charge states, but deisotoping and charge state deconvolution are often dealt with separately in many algorithms [10,16-19,24]. While high abundance charge states may be correctly detected, low abundance charge states might be missed or wrongly assigned, rendering low sensitivity results in peptide identification and inaccuracy in peptide quantification. In contrast, BPDA combines the information of isotopic peaks at different charge states as a whole to detect one peptide, lending information to better identify weak peptide signals. Thirdly, BPDA provides existence probabilities for all the peptides considered, as opposed to the fitting scores generally provided by template-matching methods, the benefits being that the existence probabilities can be directly used for probability-based evaluation of peptides and confident peptide detection similar to that of PeptideProphet [25], which is a popular software used for LC-MS/MS peptide identification. Finally, most of the parameters in the proposed method possess a clear physical meaning, since they come directly from the observation of the mass spectra. In contrast, many other approaches require the selection of numerous nonintuitive parameters, such as wavelet functions and coefficients [11-13].

## Methods

For 1 D MS spectrum, we first perform spectrum preprocessing to remove the baseline, filter the noise and generate a list of peptide candidates. Then BPDA is applied based on the developed MS model to infer the best fitting peptide signals of the observed spectrum, the results being peptide abundances, existence probabilities and so on. For 2 D LC-MS spectra, we first detect peptide elution peaks along the retention time dimension, and build elution peak groups by collecting the peaks which have similar retention time together using a method similar to [24]. Each group contains a series of consecutive spectra, which are then averaged to form a mean spectrum. The rationale of using a mean spectrum to represent the group is that the noise of consecutive spectra could be canceled out to a certain degree [11]. The BPDA algorithm is then applied to each of the mean spectra, and finally an overall peptide list is generated. The details of the preprocessing step, the proposed MS model, and the BPDA algorithm are described in the following subsections.

### Spectrum preprocessing and obtaining peptide candidates

A non-flat baseline is often observed in mass spectra, the presence of which can distort the true signal pattern. Thus the first preprocessing step is to detect and subtract the baseline from MS spectra. We use the minimum of a sliding window along the *m/z *axis as the baseline, similar to the method used in [10]. The next step is peak detection. We use the Matlab function "mspeaks" [26] to perform this task. The algorithm first identifies all local maxima in the wavelet denoised spectrum as putative peak locations. Then peaks are filtered based on their intensities and signal to noise ratios. The last step of preprocessing is to obtain a list of peptide candidates. Considering one detected peak with centroid at *m/z *value *d*, we want to find out which peptides can potentially register a peak at this position. The answer is given below in terms of the masses of such peptides:

(1) $$mass=i(d-m_{pc})-jm_{nt},\;i=1,2,\;...,\;cs,\;j=0,1,\;...,\;iso,$$

where *mass *is the mass of one peptide candidate, *m_pc _*is the mass of one positive charge and *m_nt _*is the mass shift caused by addition of one neutron. Due to mass defect, the mass shift varies for different elements. We approximate *m_nt _*using the mass shift from ^13^*C *to ^12^*C*, which is 1.0034, since Carbon contributes most to the isotope patterns. This approximation works well if the mass calibration of the instrument is correct. The parameters *cs *and *iso *are user defined maximum numbers of considered charge states and isotopic positions, respectively. It is easy to see from the above equation that each detected peak gives rise to *cs *× (*iso *+ 1) different peptide candidates (masses). These candidates exhaust all the possibilities to generate the peak with centroid *d*, but it does not follow that all the candidates really exist in the sample. Therefore, our primary goal in peptide detection is to find the existence probability of each peptide candidate. Also note that the total number of candidates should be less than or equal to *cs *× (*iso *+ 1) × number of detected peaks, as is possible that multiple peaks yield the same candidate mass.

### Modeling the mass spectrum

Suppose *N *peptide candidates are obtained from the observed spectrum using the method described in the previous section. Each candidate can generate a series of peaks over different charge states, and at each charge state several isotopic peaks can be registered. The signal generated by the *k*th peptide candidate is thus modeled by the following equation, in which *i *and *j *represent the charge state and the isotopic position of the candidate peptide, respectively:

(2) $$g_{k}(x_{m})=\sum_{i=1}^{cs}\sum_{j=0}^{iso}c_{k,ij}f(x_{m};\rho_{k,ij},\alpha_{k,ij}),\;m=1,2,\;...,\;M,$$

where the peak shape function is given by $f\left(x_{m};\rho_{k,ij},\alpha_{k,ij}\right)\;=e^{–\rho_{k,ij}{\left(x_{m}–\alpha_{k,ij}\right)}^{2}}$. That is, the peak is modeled as Gaussian-shaped, as in [27]. It is reported that the Gaussian-shaped peak approximates the reality well enough to obtain good detection results [17]. Still, this peak shape function can be adjusted for different instruments without affecting the overall structure of the algorithm.

The observed spectrum is a mixture of the signal generated by the *N *peptide candidates plus Gaussian random noise, which can be modeled as:

(3) $$y_{m}=\sum_{k=1}^{N}\lambda_{k}g_{k}(x_{m})+\epsilon_{m}=\sum_{k=1}^{N}\lambda_{k}\sum_{i=1}^{cs}\sum_{j=0}^{iso}c_{k,ij}f(x_{m};\rho_{k,ij},\alpha_{k,ij})+\epsilon_{m},\;m=1,2,\ldots ,M,$$

In the above three equations, *x_m _*is the *m*th mass-to-charge ratio (*m/z*) in the spectrum, *y_m _*is the observed intensity at *x_m_*, *M *is the number of observations, and *ϵ_m _*is Gaussian random noise with zero mean and standard deviation *σ*. The value of can be approximated by the standard deviation of the background region in the spectrum. Note that we model *ϵ_m _*as additive Gaussian which is generally a good model for the thermal noise in electronic instruments. There are reports of non-Gaussian noise in FTMS [28] and thus it is safer to apply the proposed algorithm to TOF MS instruments [29]. The parameters of the *k*th candidate, namely, *α_k,ij_*, *ρ_k,ij_*, *λ_k _*and *c_k,ij _*are discussed in detail below:

• *α_k,ij _*is the theoretic centroid (*m/z *value) of the peak generated by candidate *k*, at charge state *i *and isotopic number *j*.

(4) $$\alpha_{k,ij}=\frac{mass_{k}+i\;m_{pc}+j\;m_{nt}}{i},\;\;i=1,2,\;...,\;cs,\;j=0,1,\;...,\;iso,$$

where *mass_k _*is the mass of the *k*th candidate. Since the candidate's mass is already obtained, *α_k_*_, *ij *_can be calculated.

• *ρ_k,ij _*relates to the shape (width) of the peak centered at *α_k,ij_*. It can be estimated by using its relationship to the peak's Full Width at Half Maximum (FWHM): $\rho_{k,ij}=2\sqrt{2\ln2}/\text{FWHM}$.

• *λ_k _*is an indicator random variable, which is 1 if the *k*th peptide candidate truly exists in the sample and 0 otherwise.

• *c_k,ij _*is the height (i.e. intensity) of the peak generated by peptide *k*, at charge state *i *and isotopic number *j*.

In summary, the model considers peaks at different isotopic positions and charge states simultaneously for each peptide candidate, incorporating candidates' existence probabilities and the spectrum thermal noise.

### Bayesian peptide detection

Let

$$\theta ≜\{\lambda_{k},c_{k,ij};\;\;k=1,...,N,i=1,...,cs,j=0,...,iso\}$$

be the set of all the unknown model parameters. The goal of our algorithm is to determine the value of ***θ ***based on the observed spectrum **y **= [*y*_1_,..., *y_M _*]*^T ^*. In fact, the value of *λ_k _*is of our prime interest for the peptide detection problem. For this purpose, we can use a Bayesian approach to first obtain the *a posteriori *probability (APP) of all the parameters, *P *(***θ ***| **y**). Then the APPs *P *(*λ_k_***|y**), *k *= 1, ..., *N*, can be obtained by integration of the joint posterior distribution *P *(***θ ***| **y**) over all parameters except λ_k_. Clearly, the calculation involves high dimension integration which is not an easy task. Besides, due to the highly nonlinear nature of the data model, none of the desired APPs can be obtained analytically. To overcome the computational obstacle, we resort to the Gibbs sampling method [30], which is a variant of the Markov Chain Monte Carlo (MCMC) approach [31], to sample the model parameters.

Gibbs sampling is an iterative scheme, which uses the popular strategy of divide-and-conquer to sample a subset of parameters at a time while fixing the rest at the sample values from the previous iteration, as if they were true. In other words, for the *l*th parameter group ***θ**_l_*, we sample from the conditional posterior distribution *P*(***θ**_l_*|***θ***_-*l*_, **y**), where θ *_-l _*≜ *θ\θ_l_*. After this sampling process iterates among the parameter groups for a sufficient number of cycles (which is referred to as the "burn-in" period), convergence is reached. The samples collected afterwards are shown to be from the marginal posterior distribution *P*(***θ**_l_*|**y**), which is independent of *θ_-l_*, and thus these samples can be used to estimate the target parameters.

The Gibbs sampling process for the *k*th peptide candidate and the derivations of the conditional posterior distributions of important model parameters are briefly summarized below. The detailed derivations can be found in Additional file 1.

• **Sample the peak height vector c**_k _≜ [*c_k,ij; _i *= 1,..., *cs, j *= 0,..., *iso*]*^T ^***for the ***k***th peptide candidate**

The heights of all the possible peaks (over different charge states and isotopic positions) of the *k*th peptide candidate are included in the peak height vector ***c**_k _*and are sampled simultaneously from the conditional posterior distribution of ***c**_k_*, which, by the Bayesian principle, is proportional to the likelihood times the prior:

(5) $$P(c_{k}|\text{y},\theta_{-{\text{c}}_{k}})\propto P(y|\theta )P(c_{k}),$$

where $\theta_{-{\text{c}}_{k}}≜\theta \backslash c_{k}$.

The derivations of the likelihood, the prior distribution and the conditional posterior distribution of ***c****_k _*are given in Additional file 1.

• Sample the peptide existence indicator variable *λ_k_*

The conditional posterior distribution of *λ_k _*is given by

$$P(\lambda_{k}|\text{y},\theta_{-\lambda_{k}})\;\propto \;p(\text{y}|\theta )p(\lambda_{k})$$

where $\theta_{-\lambda_{k}}≜\theta \backslash \lambda_{k}$.

The derivations of the likelihood, the prior distribution and the conditional posterior distribution of *λ_k _*are given in Additional file 1

The complexity of the proposed Gibbs sampling algorithm is determined by two factors: (1) the sheer number of peptide candidates, and (2) the correlation between parameters that need to be sampled. The algorithm complexity grows exponentially with the number of peptide candidates, and the correlation between parameters reduces the sampling efficiency. To address these two issues, we first partition non-overlapping peptide candidates into different groups. The proposed algorithm can be applied to each group in a parallel manner and the algorithm complexity is reduced, because within each group the number of candidates is reduced, and the corresponding signal-containing spectrum region is restricted. Peptide candidates within each group are then clustered by the k-means clustering algorithm [32], the distance measure being the correlation between peptide candidate signals. Peptide candidates within a cluster have strong correlations among each other, and their indicator variables are sampled from the joint conditional posterior distribution. These two measures improve the overall efficiency of the algorithm. The pseudocode of the entire Gibbs sampling process is given in Additional file 2: Table S1.

The samples taken after convergence can be used to estimate the target parameters. Particularly, the existence probability of peptide *k *is calculated as

(6) $$P(\lambda_{k}=1|y)=\frac{1}{R-r_{0}+1}\sum_{r=r_{0}}^{R}\lambda_{k}^{r},$$

where *r*_0 _is the first iteration after convergence is reached, *R *is the total number of iterations, and $\lambda_{k}^{r}$ is the sample value of *λ_k _*in the *r*th iteration. The *k*th peptide candidate is said to be detected if its existence probability *P*(*λ_k _*= 1|**y**) is greater than a predefined threshold.

## Results

We report below the observed performance of BPDA, side by side with well-known tools, such as OpenMS and Decon2LS, in a number of experiments using both synthetic and real data.

### Synthetic data

It is difficult to evaluate the performance of a given detection method using real data due to the existence of unpredictable contaminants and the unknown true composition of the samples. The merit of using simulated data is that the ground truth is known and thus algorithm evaluation can be carried out [27,29].

#### Synthetic 20-mix spectra with different abundance levels (SNRs)

**Figure 1:**
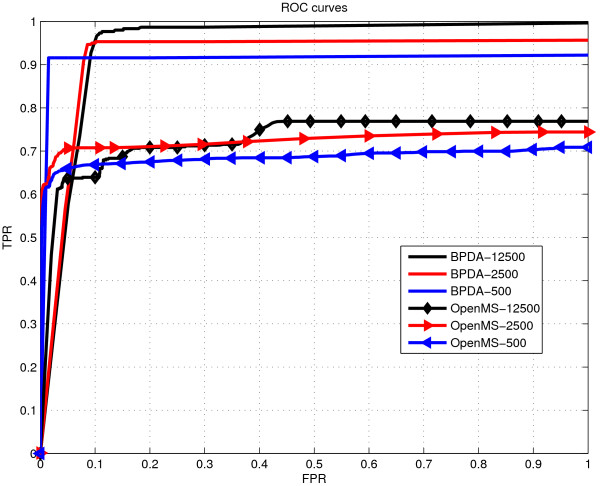
**ROC results for synthetic 20-mix spectra with different abundance levels (SNRs)**. ROC results for synthetic 20-mix spectra with different abundance levels *a *= 500, 2500 and 12500.

First, to test the robustness of our algorithm, we generated MS data sets with different signal to noise ratios (SNRs), using the method described in [27]. In fact, the mean signal strength (i.e., peptide abundance) was varied while the noise level (i.e., the mean and variance of the noise) was fixed. For each peptide abundance level *a*, *a *∈ {500, 2500, 12500}, the simulation was repeated 50 times. In each repetition, 20 true peptides (with abundance level *a *and masses randomly selected from a quality-control Shewanella Oneidensis data set provided by PNNL [33]) served as the input of the data model given by Eq. 3. The charge state distribution of one peptide was modeled by a binomial distribution, which was reported to approximate the real data well [27]. The isotopic distribution was obtained for each peptide by using the Averagine model [22] and the Mercury algorithm [21]. The output consists of a simulated mass spectrum. BPDA was applied to obtain the peptide existence probabilities and abundance results. Its performance was evaluated by the classic Receiver Operating Characteristic (ROC) curve. To obtain the ROC curve, first a series of detection levels τ ranging from 0 to 1 with 0.001 increments was selected. Peptides with existence probabilities not less than τ were said to be detected at this specific detection level. The True Positive Rate (TPR) and False Positive Rate (TPR) were then calculated at each detection level as follows:

$TPR=\frac{TruePositive}{TruePositive+FalseNegative}$ and $FPR=\frac{FalsePositive}{FalsePositive+TrueNegative}$. One ROC curve (each point on the curve was a pair of TPR and FPR at one detection level) was plotted for each repetition. And the averaged ROC curve for one abundance level was obtained by averaging all the ROC curves corresponding to the same abundance level. We also applied OpenMS on the same data sets -- to do so, we first wrote the simulated MS data into a text file with three columns specified by elution time, *m/z*, and intensity, respectively. Next, the text file was converted to mzXML (which is a valid input file format for OpenMS) by the FileConverter tool integrated in the OpenMS software package [34]. Finally, OpenMS was applied on the mzXML file to give the detection results including detected features and their qualities. The ROC results given by the two algorithms for different abundance levels are shown in Figure 1.

#### Synthetic 10-mix spectrum with overlapping peptides

**Figure 2:**
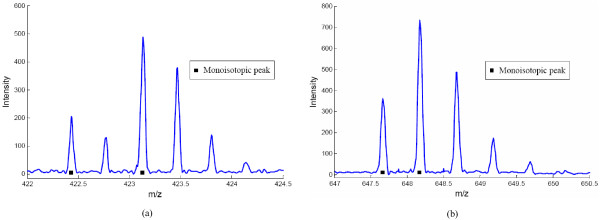
**Illustration of overlapping peptides observed in the synthetic 10-mix spectrum**. (a) Overlapping peptide signals observed in *m/z *range 422-424.5, which is generated by monoisotopic masses 1264.279 and 1266.383 at charge state 3. OpenMS missed the first one while BPDA detected both. (b) Overlapping peptide signals observed in *m/z *range 647-650.5, which is generated by monoisotopic masses 1293.323 and 1294.345 at charge state 2. OpenMS missed the first one while BPDA detected both.

As noted before, overlapping peptide peaks can complicate the mass spectra and make the detection problem much harder. Thus, we investigated the performance of BPDA in the presence of overlapping peptides. A simulated 10-mix spectrum was generated by 5 pairs of overlapping peptides with unique masses: 1264.279, 1266.383, 1382.247, 1388.367, 1293.323, 1294.345, 1312.441, 1313.451, 1327.386 and 1329.378 Da. The detection results for the comparison between BPDA and OpenMS are summarized in Table 1. BPDA detected all 10 peptides when *FPR *= 0.1, with very small mass deviations and quite accurate abundance results. Almost all charge states of the 10 true peptides were correctly reported, except for the highest charge state of the 5th and the 9th peptides. These two charge states were missed because the corresponding peptide signal was very weak. In contrast, when *FPR *= 0.1, OpenMS only detected the 3rd, the 7th and the 9th peptides. And when FPR increased to 0.3, OpenMS achieved its highest TPR (0.6). But it could detect only one pair of peptides (the one with the least overlap) and missed one peptide in each of the other 4 pairs. Two examples are given in Figure 2 to illustrate the observed overlapping peptide signals and the detection results. The abundance results given by OpenMS were not close to those of the true peptides (although the total abundance of each overlapping pair was not far away from the corresponding total abundance of the true peptides). In total, 18 out of 36 charge states were correctly detected by OpenMS for the 10 peptides, while BPDA correctly detected 34 out of 36, a much larger number.

**Table 1:** Results for synthetic 10-mix spectrum with overlapping peptides

	BPDA	OpenMS
True Mass (Da)/Intn/CS	dM (Da)/Intn/CS	dM (Da)/Intn/CS
1264.279/0.034/1-3	-0.0065/0.032/1-3	NA

1266.383/0.103/1-3	-0.0025/0.110/1-3	-0.0025/0.156/1-3

1382.247/0.171/1-4	0.0028/0.181/1-4	0.0031*/0.228/1-3

1388.367/0.114/1-4	-0.0073/0.097/1-4	-0.0046/0.150/1-3

1293.323/0.006/1-3	-0.0081/0.007/1-2	NA

1294.345/0.008/1-3	-0.0124/0.008/1-3	0.0033/0.018/1-2

1312.441/0.229/1-4	0.0018/0.247/1-4	0.0019*/0.334/1-4

1313.451/0.183/1-4	-0.0061/0.173/1-4	NA

1327.386/0.080/1-4	-0.0035/0.067/1-3	0.0061*/0.114/1-3

1329.378/0.072/1-4	-0.0035/0.078/1-4	NA

Results for the 10-mix data set. Intn, CS and dM denote the normalized intensity, detectable charge states and the mass deviation from the true mass, respectively. When FPR = 0.1, BPDA was able to detect all 10 true peptides, while OpenMS detected only 3 peptides (marked by *). OpenMS achieved its highest TPR (0.6) when FPR = 0.3.

We remark that Decon2LS results are missing from both synthetic experiments described previously because the synthetic data could not be loaded, causing the program to crash (the data was contained in a mzXML file converted from a 3-column text file by the OpenMS FileConverter tool, whose format was successfully verified against mzXML version 2.1). We contacted Decon2LS's developers, but did not hear from them in time to have the Decon2LS results included.

### Real data

In this section we report results from experiments carried out with real MS data. The test data and parameter files used for different software tools were provided as supplementary files on the BPDA project website. We stick mainly to the recommended parameter values while only adjusted a few parameters such as mass range and detection level to adapt to each data set.

#### MALDI-TOF MS 7-mix spectrum

We tested BPDA on MALDI-TOF MS 7-mix spectrum, which contained seven standard peptides with monoisotopic masses 1045.535, 1295.678, 1346.728, 1618.815, 2092.079, 2464.191 and 3146.464 Dalton [35]. The spectrum was collected on a Bruker ultraFlex MALDI TOF in the reflectron mode. As stated before, MALDI mostly generates singly charged ions, so we only considered charge state 1 in the test. Since there were contaminants in the data set, the goal was to check whether a detection algorithm could find all the seven true peptides. The detection results of BPDA, Decon2LS, OpenMS, and the commercial software flexAnalysis developed by Bruker Daltonics [36] are summarized in Table 2. BPDA detected the first six peptides with a mean (absolute) mass deviation 0.018 Da. Decon2LS missed the fifth and the last peptides, and the five detected peptides were of a mean mass deviation 0.013 Da. OpenMS missed the forth and the last peptides, and the five detected peptides were of a mean mass deviation 0.025 Da. The commercial software flexAnalysis missed the fifth and the last peptides, and the five detected peptides were of a mean mass deviation 0.013 Da. It can be seen that for the detected peptides, the four algorithms yielded similar intensity results. Only BPDA and OpenMS were able to detect the fifth peptide which had the lowest abundance among the first six peptides. And all methods failed to report the last peptide. Visual inspection suggested that this peptide generated very weak signal and its abundance was about one third of the fifth peptide.

**Table 2:** Results for the MALDI-TOF MS 7-mix spectrum

	BPDA	OpenMS	Decon2LS	Bruker
**True Masses (Da)**	**dM (Da)/Intn**	**dM (Da)/Intn**	**dM (Da)/Intn**	**dM (Da)/Intn**

1045.535	-0.023/0.550	0.019/0.655	-0.021/0.615	-0.023/0.532

1295.678	0.003/0.173	0.026/0.232	0.002/0.168	-0.001/0.167

1346.728	0.017/0.053	0.040/0.070	0.013/0.050	0.011/0.052

1618.815	0.035/0.178	NA	0.024/0.137	0.022/0.202

2092.079	0.021/0.004	0.021/0.009	NA	NA

2464.191	-0.012/0.042	0.020/0.034	-0.007/0.030	-0.009/0.047

3146.464	NA	NA	NA	NA

Results for the 7-mix data set. Intn and dM denote the normalized intensity, and the mass deviation from the true mass, respectively.

#### High-resolution LC-MS data set MyoLCMS

The preparation of the MyoLCMS data set is detailed as below: the data set was collected from an overnight tryptic digest of horse myoglobin. Capillary liquid chromatography-mass spectrometry (cLC/MS) was performed with a splitless nanoLC-2 D pump (Eksigent), a 50 mm-i.d. column packed with 10 cm of 5 mm-o.d. C18 particles, nanoelectrospray and a high-resolution time-of-flight mass spectrometer (MicrOTOF; Bruker Daltonics). The cLC gradient was 2 to 98% 0.1% formic acid/acetonitrile in 172 seconds at 400 nL/min. Sample was injected at a concentration of 60 fmol/mL with an injection volume of 10 mL (600 fmol injected on-column).

**Figure 3:**
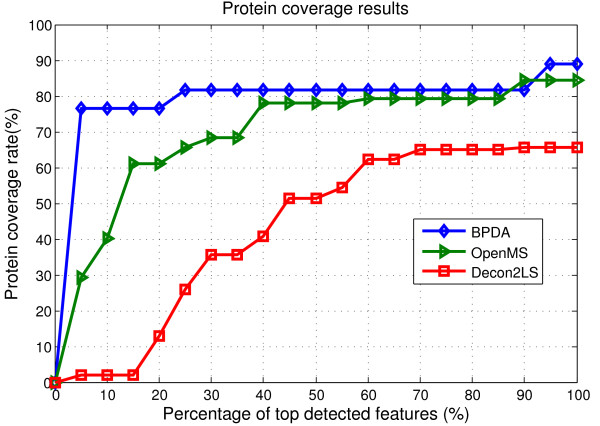
**Results for the LC-MS data set MyoLCMS**. Protein coverage results achieved by BPDA, OpenMS, and Decon2LS.

There were 172 spectra with a *m/z *range 44.9 to 3005. To apply BPDA, we first grouped peptide elution peaks, as described in the Method section. A total of 17 groups were obtained, each containing 10-20 consecutive spectra. A mean spectrum was generated for each group, and BPDA was then applied. The detection results of BPDA, OpenMS, and Decon2LS, which was applied in conjunction with VIPER [37], are summarized in Additional file 3: Table S2 (we also considered the method implemented in the SpecArray package [15], but found it to be inferior to BPDA, OpenMS, and Decon2LS -- the results were then omitted for the sake of conciseness). The number of features with unique monoisotopic masses detected by BPDA, OpenMS, and Decon2LS-Viper were 1635, 2176 and 823, respectively. In fact, it is not very informative to evaluate the performance of a detection algorithm solely based on the number of detected features, because of the presence of contaminants and false positive detections. Therefore, we focus on the top detected features yielded by each detection algorithm. Detected features were ranked by quality in descending order. Different algorithms utilize different quality metrics; for example, Decon2LS and OpenMS provide a quality score which measures how well an observed isotope pattern matches the predicted isotope pattern, while BPDA provides the peptide existence probability (see Eq. 6) as the quality measure. For each detection algorithm, for a given percentage of top detected features, we calculated the number of detected horse myoglobin peptides and the protein coverage rate. Note that by in-silico digestion of horse myoglobin, there are 39 tryptic peptides with less than 2 missed cleavage sites (19 of which do not contain any missed cleavage sites). Ideally, we should compare algorithms with known peptide composition in the sample and report protein coverage at different false positive rates. However, due to possible peptide contamination in the sample in any LC/MS experiment, actual peptide species presented in the sample are never known and this prevents us from estimating the false positive rates on the reported peptide list. As a result, the statistical significance of reported peptides by different peptide identification algorithms cannot be evaluated and the only option left for users in hope of obtaining a list of peptides with relatively low false positive rate is by applying a percentage threshold on the quality score reported by different algorithms. Thus, protein coverage v.s. percentage threshold on quality score is a meaningful measurement of the performance of peak detection algorithms and the results are shown in Figure 3. We need to point out that although the protein coverage of OpenMS seems to be comparable with the proposed algorithm in regions where the quality score percentage threshold is large, in such regions the reported peptide list may contain a lot of false positives and it is not an indication of good or bad algorithm performance. Instead, how quickly an algorithm reaches high protein coverage as the percentage threshold increases should be the measurement of the performance. In Figure 3, we can see that BPDA reaches high protein coverage much faster than other algorithms at low percentage threshold regions.

## Discussion

We observed in our experiments that BPDA performs well on both simulated data and real data, for various SNRs and resolutions, and in complex cases where features overlap.

For the synthetic 20-mix experiment, we observe in Figure 1 that the sensitivity (i.e., TPR) of BPDA was consistently higher than that of OpenMS for each abundance level, and both methods gave better sensitivity results as the abundance level (i.e., SNR) increased. Also it is observed that BPDA was quite robust for different SNRs. For the synthetic 10-mix experiment with overlapping peptides, we saw that BPDA detected all the peptides at a small false-positive rate *FPR *= 0.1, with very small mass deviations and quite accurate abundance results, and nearly all the charge states of the 10 true peptides were correctly reported. In contrast, at *FPR *= 0.1, OpenMS could detect only a few of the peptides. The abundance results given by OpenMS were not very close to those of the true peptides. Also OpenMS could only detect about half of the charge states.

The results obtained with real data corroborated the findings made with the synthetic experiments. For the MALDI-TOF MS 7-mix data, the four algorithms yielded similar intensity results, but BPDA was the only one to detect six out of the seven peptides. For the MyoLCMS experiment, we focused on protein coverage results, which is an important criterion to determine the confidence in protein identification and quantification [38,39]. It was observed that BPDA displayed the largest protein coverage among the programs tested.

## Conclusions

We have presented BPDA, a Bayesian approach for peptide detection. Feature extraction in MS analysis is difficult because peptides can register multiple peaks. We model peptide signals based on both charge state distributions and isotopic distributions. And unlike perviously published methods, where the detection only utilizes isotopic distributions and works at each single charge state alone, BPDA takes into account the charge state distribution as well, and performs deisotoping and charge state deconvolution at the same time, thus lending information to better identify weak peptide signals and produce more robust results. Moreover, the proposed approach is systematic. It is based on a rigorous statistical framework and avoids problems, such as voting, thresholding and matching ambiguities, generally encountered in algorithms based on template matching.

We have shown that BPDA performs well on both simulated data and real data, for various SNRs and resolutions, and in complex cases where features overlap. Our experimental results indicate that BPDA compares very favorably with commercial software flexAnalysis and commonly used open-source softwares such as OpenMS and Decon2LS in terms of detection performance. As for computational time, BPDA is a global-based approach, which looks for the optimal solution iteratively through Gibbs sampling, while template-matching based algorithms such as OpenMS and Decon2LS work on a local region at a time and calculate the fitting score, which typically does not require much computation. Hence, BPDA is expected to be more time-consuming than those algorithms, especially when running under the raw data mode. For example, for 10-mix data set, the running times for OpenMS and BPDA were 1 minutes and 30 minutes, respectively -- although these figures are not entirely comparable at present, since OpenMS and Decon2LS are developed using the C programming language, while BPDA is developed using Matlab; we plan to translate the Matlab code into C in future work. In addition, we point out that the user can choose the centroid mode to run BPDA as a tradeoff between running time and performance.

## Availability and requirements

**Project name: **BPDA

**Project home page: **http://gsp.tamu.edu/Publications/supplementary/sun10a/bpda

**Operating system(s): **Platform independent

**Programming language: **Matlab

**Licence: **GNU GPL (note that we do not allow material transfer agreements or software transfer agreements for academics)

**Any restrictions to use by non-academics: **licence needed.

## Authors' contributions

YS developed and implemented the algorithm, conducted all simulations and wrote the initial draft of the paper. JZ conceived the algorithm, advised YS on algorithm development and revised the paper. UBN advised YS on the numerical experiments and revised the paper. ERD revised the paper.All authors read and approved the final manuscript.

## Supplementary Material

Additional file 1 Suppl file 1: Detailed derivations of the likelihood, the prior distributions and the conditional posterior distributions of model parameters. Click here for file

Additional file 2 Table S1: The pseudocode of the Gibbs sampling process. Click here for file

Additional file 3 Table S2: Detection results for high-resolution LC-MS data set MyoLCMS. Click here for file
