# Supplementary material for: BPDA - A Bayesian peptide detection algorithm for mass spectrometry
Source: BMC Bioinformatics. 2010 Sep 29;11:490. doi: 10.1186/1471-2105-11-490 (PMC3098078; doi:10.1186/1471-2105-11-490)
Supplement: Additional file 1 — Suppl file 1: Detailed derivations of the likelihood, the prior distributions and the conditional posterior distributions of model parameters. [file 1471-2105-11-490-S1.PDF]

Supplementary file for "BPDA – A Bayesian peptide detection algorithm for mass spectrometry"

Youting Sun[1], Jianqiu Zhang*[2] , Ulisses Braga-Neto[1] and Edward R. Dougherty*[1,3,4]

[1]Department of Electrical and Computer Engineering, Texas A&M University, College Station, TX 77843, USA
[2]Department of Electrical and Computer Engineering, University of Texas at San Antonio, San Antonio, TX 78249, USA
[3]Computational Biology Division, Translational Genomics Research Institution, Phoenix, AZ 85004, USA
[4]Department of Bioinformatics and Computational Biology, University of Texas M.D. Anderson Cancer Center, Houston, TX 77030, USA

Email: Youting Sun - charonsun@tamu.edu; Jianqiu Zhang*- Michelle.Zhang@utsa.edu; Ulisses Braga-Neto - ulisses@ece.tamu.edu; Edward R. Dougherty*- edward@ece.tamu.edu;

*Corresponding author

Bayesian peptide detection

Let

$$\boldsymbol{\theta} \triangleq \{\lambda_k, c_{k,ij}; k = 1, \ldots, N, \; i = 1, \ldots, cs, \; j = 0, \ldots, iso\}$$

be the set of unknown model parameters. Given the observed spectrum $\mathbf{y} = [y_1, \ldots, y_M]^T$, we apply Gibbs sampling [1] to determine the value of $\boldsymbol{\theta}$. Gibbs sampling uses the popular strategy of divide-and-conquer to sample a subset of parameters at a time while fixing the rest at the sample values from the previous iteration, as if they were true. In other words, for the l-th parameter group $\boldsymbol{\theta}_l$, we sample from the conditional posterior distribution $P(\boldsymbol{\theta}_l|\boldsymbol{\theta}_{-l}, \mathbf{y})$, where $\boldsymbol{\theta}_{-l} \triangleq \boldsymbol{\theta} \setminus \boldsymbol{\theta}_l$, with values obtained from the previous iteration. After this sampling process iterates among the parameter groups for a sufficient number of cycles (i.e., the "burn-in" period), convergence is reached. The samples collected afterwards are shown to be from the marginal posterior distribution $P(\boldsymbol{\theta}_l|\mathbf{y})$ which is independent of $\boldsymbol{\theta}_{-l}$, and thus these samples can be used to estimate the target parameters.

The Gibbs sampling process for the kth peptide candidate and the derivations of the conditional posterior distributions of model parameters are given below.

- **Sample the peak height vector $\mathbf{c}_k \triangleq [c_{k,ij}; i = 1, \ldots, cs, \; j = 0, \ldots, iso]^T$ for the kth peptide candidate**

By the Bayesian principle, the conditional posterior distribution of $\mathbf{c}_k$ is proportional to the likelihood times the prior, that is,

$$P(\mathbf{c}_k\,|\mathbf{y},\boldsymbol{\theta}_{-\mathbf{c}_k}) \propto P(\mathbf{y}|\boldsymbol{\theta})\text{Prior}(\mathbf{c}_k), \tag{1}$$

where $\boldsymbol{\theta}_{-\mathbf{c}_k} \triangleq \boldsymbol{\theta} \setminus \mathbf{c}_k$.

It is easy to show the likelihood satisfies

$$P(\mathbf{y}|\boldsymbol{\theta}) \propto \exp\{-\frac{1}{2\sigma^2}(\mathbf{y}-\mathbf{G}\lambda^{(0)}-\lambda_k\mathbf{H}_k\mathbf{c}_k)^T\mathbf{I}_{M\times M}(\mathbf{y}-\mathbf{G}\lambda^{(0)}-\lambda_k\mathbf{H}_k\mathbf{c}_k)\}, \tag{2}$$

where

$$\boldsymbol{\lambda}^{(s)} \triangleq [\lambda_1,\ldots,\lambda_k=s,\ldots,\lambda_N]^T,\ s\in\{0,1\}, \tag{3}$$

$$\mathbf{G} = \begin{pmatrix} g_1(x_1) & g_2(x_1) & \ldots & g_N(x_1) \\ g_1(x_2) & g_2(x_2) & \ldots & g_N(x_2) \\ \vdots & \vdots & \ddots & \vdots \\ g_1(x_M) & g_2(x_M) & \ldots & g_N(x_M) \end{pmatrix}_{M\times N}, \tag{4}$$

with the (m,k)-th entry $g_k(x_m)=\sum_{i=1}^{cs}\sum_{j=0}^{iso}c_{k,ij}f(x_m;\rho_{k,ij},\alpha_{k,ij})$ representing the signal at m/z value x_m generated by peptide candidate k. In addition, $\mathbf{H}_k=[h_{m,(i-1)\times(iso+1)+j+1}]_{M\times cs(iso+1)}$, with $h_{m,(i-1)\times(iso+1)+j+1}=f(x_m;\rho_{k,ij},\alpha_{k,ij})=e^{-\rho_{k,ij}(x_m-\alpha_{k,ij})^2}$.

The heights of the isotopic peaks of peptide candidate k at charge state i follow a multinomial distribution [2], which by the Central Limit Theorem can be approximated by a Gaussian distribution as below:

$$\begin{aligned} P(c_{k,ij}, j=0,\ldots,iso\,|a_k,\eta_{k,i},\boldsymbol{\pi}_k) &= MN(a_k\eta_{k,i},\boldsymbol{\pi}_k) && (5)\\ &\approx N(a_k\eta_{k,i}\boldsymbol{\pi}_k, a_k\eta_{k,i}[\text{diag}(\boldsymbol{\pi}_k)-\boldsymbol{\pi}_k^T\boldsymbol{\pi}_k]), && (6)\end{aligned}$$

where a_k is the total centroid intensity of candidate k, and $\boldsymbol{\eta}_k \triangleq [\eta_{k,1},\eta_{k,2},\ldots,\eta_{k,cs}]^T$ and $\boldsymbol{\pi}_k \triangleq [\pi_{k,0},\pi_{k,1},\ldots,\pi_{k,iso}]^T$ denote the charge state distribution and the theoretical isotopic distribution of peptide candidate k, respectively.

Thus the prior distribution of the peak height vector $\mathbf{c}_k$ is given by:

$$\text{Prior}(\mathbf{c}_k) = P(\mathbf{c}_k\,|a_k,\boldsymbol{\eta}_k,\boldsymbol{\pi}_k) \approx N(\boldsymbol{\mu}_{\mathbf{c}_k},\boldsymbol{\Sigma}_{\mathbf{c}_k}), \tag{7}$$

where

$$\boldsymbol{\mu}_{\mathbf{c}_k} = [a_k\eta_{k,1}\boldsymbol{\pi}_k^T, a_k\eta_{k,2}\boldsymbol{\pi}_k^T, \ldots, a_k\eta_{k,cs}\boldsymbol{\pi}_k^T]^T, \tag{8}$$

$$\boldsymbol{\Sigma}_{\mathbf{c}_k} = \text{diag}(\Sigma_i), \tag{9}$$

with

$$\Sigma_i = a_k\eta_{k,i}[\text{diag}(\boldsymbol{\pi}_k) - \boldsymbol{\pi}_k^T\boldsymbol{\pi}_k],\ i = 1, 2, \ldots, cs. \tag{10}$$

Substituting Eq. 2 and Eq. 7 into Eq. 1 and it can be shown by algebraic manipulations [3] that the conditional posterior distribution of $\mathbf{c}_k$ is also Gaussian, with the mean vector and covariance matrix given below:

$$\boldsymbol{\Sigma}_{\mathbf{c}_k|\mathbf{y},\boldsymbol{\theta}_{-\mathbf{c}_k}} = (\mathbf{I} - \mathbf{K}\mathbf{H}_k)\boldsymbol{\Sigma}_{\mathbf{c}_k}, \tag{11}$$

$$\boldsymbol{\mu}_{\mathbf{c}_k|\mathbf{y},\boldsymbol{\theta}_{-\mathbf{c}_k}} = \boldsymbol{\mu}_{\mathbf{c}_k} + \mathbf{K}(\mathbf{y} - \mathbf{G}\lambda^{(0)} - \mathbf{H}_k\boldsymbol{\mu}_{\mathbf{c}_k}), \tag{12}$$

where $\mathbf{K} \triangleq \boldsymbol{\Sigma}_{\mathbf{c}_k}\mathbf{H}_k^T\left(\mathbf{H}_k\boldsymbol{\Sigma}_{\mathbf{c}_k}\mathbf{H}_k^T + \sigma^2\mathbf{I}_{M\times M}\right)^{-1}$ is known as the Kalman gain matrix [4].

- **Sample a_k, the total centroid intensity of candidate k**

  The conditional distribution of a_k takes different forms for different values of λ_k.

  When $\lambda_k = 1$ (the kth candidate is inferred to be present), by definition,

$$a_k\,|(c_{k,ij}, \lambda_k = 1) = \sum_{i=1}^{cs}\sum_{j=0}^{iso} c_{k,ij}. \tag{13}$$

  When $\lambda_k = 0$ (the kth candidate is inferred to be absent), the distribution of a_k, which is independent of the observation $\mathbf{c}_k$, is modeled by a uniform distribution as below:

$$P(a_k\,|c_{k,ij}, \lambda_k = 0) = \text{Unif}(0, u_k), \tag{14}$$

  where u_k is the upper bound of a_k.

- **Sample $\boldsymbol{\eta}_k \triangleq [\eta_{k,1}, \eta_{k,2}, \ldots, \eta_{k,cs}]^T$, the charge state distribution of peptide candidate k**

  Unlike the isotopic distribution, the charge state distribution cannot be theoretically predicted even when the peptide sequence is given. Thus $\boldsymbol{\eta}_k$ needs to be estimated by the Gibbs sampling process.

Let $\boldsymbol{b}_k \triangleq [b_{k,1}, b_{k,2}, \ldots, b_{k,cs}]^T$, where $b_{k,i}$ is the total centroid abundance of peptide k at charge state i. Given the charge state distribution and the total centroid abundance of peptide k, the likelihood of $\boldsymbol{b}_k$ is multinomial:

$$P(\boldsymbol{b}_k|\boldsymbol{\eta}_k, a_k) = \text{MN}(a_k, \boldsymbol{\eta}_k). \tag{15}$$

As is well known, the conjugate prior to a multinomial likelihood is Dirichlet, which is also a reasonable choice for the prior of $\boldsymbol{\eta}_k$. Thus, let the prior of $\boldsymbol{\eta}_k$ be a Dirichlet distribution with parameter $w\boldsymbol{\alpha}$, where w is a weight parameter that controls the strength of the prior information. A small w is preferable if uncertainty resides in the prior, and vice versa. Then the posterior distribution of $\boldsymbol{\eta}_k$ is given by

$$\begin{aligned} P(\boldsymbol{\eta}_k\,|\mathbf{b}_k\,) &\propto P(\mathbf{b}_k\,|\boldsymbol{\eta}_k\,)\text{Prior}(\boldsymbol{\eta}_k) && (16)\\ &= \text{Dirichlet}(w\boldsymbol{\alpha} + \mathbf{b}_k). && (17)\end{aligned}$$

- **Sample the peptide existence indicator variable λ_k**

The conditional posterior distribution of λ_k is given by

$$\begin{aligned} P(\lambda_k\,|\mathbf{y}, \boldsymbol{\theta}_{-\lambda_k}\,) &\propto P(\mathbf{y}\,|\theta\,)\text{Prior}(\lambda_k)\\ &\propto \exp\{-\frac{1}{2\sigma^2}\|\mathbf{y} - \mathbf{G}\boldsymbol{\lambda}\|^2\}\text{Prior}(\lambda_k), && (18)\end{aligned}$$

where $\mathbf{G}$ is defined in Eq. 4.

The log-likelihood ratio (LLR) of λ_k can be calculated as below

$$\begin{aligned} LLR_{\lambda_k} &= \ln\frac{P(\lambda_k = 1\,|\mathbf{y}, \boldsymbol{\theta}_{-\lambda_k}\,)}{P(\lambda_k = 0\,|\mathbf{y}, \boldsymbol{\theta}_{-\lambda_k}\,)}\\ &= -\frac{1}{2\sigma^2}(\|\mathbf{y} - \mathbf{G}\boldsymbol{\lambda}^{(1)}\|^2 - \|\mathbf{y} - \mathbf{G}\boldsymbol{\lambda}^{(0)}\|^2) + \ln\frac{P(\lambda_k = 1)}{P(\lambda_k = 0)}, && (19)\end{aligned}$$

where $\boldsymbol{\lambda}^{(s)}, s \in \{0, 1\}$ is defined by Eq. 3.

If no prior knowledge is available about which peptide candidates are more likely to be present in the sample, then a reasonable choice for the prior of λ_k could be the uniform distribution. Therefore the last term in Eq. 19 can be dropped. The conditional posterior distribution of λ_k is then obtained based on the log-likelihood ratio as follows:

$$P(\lambda_k = 1 \,|\mathbf{y}, \boldsymbol{\theta}_{-\lambda_k}) \;=\; \frac{1}{1+e^{-LLR_{\lambda_k}}}, \tag{20}$$

$$P(\lambda_k = 0 \,|\mathbf{y}, \boldsymbol{\theta}_{-\lambda_k}) \;=\; 1 - P(\lambda_k = 1 \,|\mathbf{y}, \boldsymbol{\theta}_{-\lambda_k}). \tag{21}$$

References

1. Geman S, Geman D: **Stochastic relaxation, Gibbs distributions, and the Bayesian restoration of images**. *IEEE Trans. Pattern Anal. Mach. Intell.* 1984, **6**:721–741.
2. Kaur P, O'Connor PB: **Use of statistical methods for estimation of total number of charges in a mass spectrometry experiment**. *Analytical Chemistry* 2004, **76**:2756–2762.
3. Anderson BDO, Moore JB: *Optimal filtering*. Englewood Cliffs, NJ, USA: Prentice-Hall 1979.
4. Burgers G, Leeuwen PJ, Evensen G: **Analysis scheme in the ensemble Kalman filter**. *Monthly Weather Review* 1998, **126**:1719–1724.
