# Supplementary material for: BPDA - A Bayesian peptide detection algorithm for mass spectrometry
Source: BMC Bioinformatics. 2010 Sep 29;11:490. doi: 10.1186/1471-2105-11-490 (PMC3098078; doi:10.1186/1471-2105-11-490)
Supplement: Additional file 2 — Table S1: The pseudocode of the Gibbs sampling process. [file 1471-2105-11-490-S2.PDF]

**Table S1 - The Gibbs sampling process**

| |
|---|
| 1. Partition peptide candidates into $G$ non-overlapping groups. |
| 2. For group $g = 1 \, \text{to} \, G$ |
| 3. Extract the signal-containing spectrum region. |
| 4. Cluster candidates into $S$ clusters. |
| 5. For iteration $r = 1 \, \text{to} \, R$ |
| 6. For cluster $s = 1 \, \text{to} \, S$ |
| 7. For peptide candidate $k = i_1^s \, \text{to} \, i_{N_s}^s$ |
| 8. Draw $\mathbf{c}_k^r$ based on its conditional posterior distribution. |
| 9. end of k loop |
| 10. Draw $\lambda_k^r, k = i_1^s \, \ldots, \, i_{N_s}^s$ for the cluster according to the joint conditional posterior distribution. |
| 11. end of $s$ loop |
| 12. end of $r$ loop |
| 13. end of $g$ loop |
