# Supplementary material for: BPDA - A Bayesian peptide detection algorithm for mass spectrometry
Source: BMC Bioinformatics. 2010 Sep 29;11:490. doi: 10.1186/1471-2105-11-490 (PMC3098078; doi:10.1186/1471-2105-11-490)
Supplement: Additional file 3 — Table S2: Detection results for high-resolution LC-MS data set MyoLCMS. [file 1471-2105-11-490-S3.PDF]

Table S2 - Results for high-resolution LC-MS data set MyoLCMS

	BPDA	OpenMS	Decon2LS
Number of detected monoisotopic masses (features)	1635	2176	823
Average number of charge states for each monoisotopic mass	2.40	1.28	NA
Protein coverage of the top 5% detected features (%)	76.6	29.2	2.0
Protein coverage of the top 40% detected features (%)	81.8	77.9	40.9
No. of horse myoglobin peptides reported in the top 5% detected features	15	3	1
No. of horse myoglobin peptides reported in the top 40% detected features	16	11	7
Mean mass deviation of horse myoglobin peptides in the top 5% detected features (Da)	0.004	0.019	0.020
Mean mass deviation of horse myoglobin peptides in the top 40% detected features (Da)	0.004	0.014	0.014
